# Combined impact of Medicare’s hospital pay for performance programs on quality and safety outcomes is mixed

**DOI:** 10.1186/s12913-022-08348-w

**Published:** 2022-07-28

**Authors:** Teresa M. Waters, Natalie Burns, Cameron M. Kaplan, Ilana Graetz, Joseph Benitez, Roberto Cardarelli, Michael J. Daniels

**Affiliations:** 1 Department of Health Management and Policy, University of Kentucky College of Public Health, 111 Washington, Avenue, KY 40536 Lexington, USA; 2 Department of Statistics, University of Florida, 102 Griffin-Floyd Hall, PO Box 118545, 32611 Gainesville, FL USA; 3 Department of Medicine, University of Southern California Keck School of Medicine, 2020 Zonal Ave. IRD 306, 90089 Los Angeles, CA USA; 4 Department of Health Policy and Management, Emory University School of Public Health, 1518 Clifton Road NE, 30322 Atlanta, GA USA; 5 Department of Family Medicine, University of Kentucky College of Medicine, 2195 Harrodsburg Road, Suite 125, 40504 Lexington, KY USA

**Keywords:** Pay-for-performance, Patient safety, Quality indicators, Hospitals, Medicare

## Abstract

**Background:**

Three major hospital pay for performance (P4P) programs were introduced by the Affordable Care Act and intended to improve the quality, safety and efficiency of care provided to Medicare beneficiaries. The financial risk to hospitals associated with Medicare’s P4P programs is substantial. Evidence on the positive impact of these programs, however, has been mixed, and no study has assessed their combined impact. In this study, we examined the combined impact of Medicare’s P4P programs on clinical areas and populations targeted by the programs, as well as those outside their focus.

**Methods:**

We used 2007–2016 Healthcare Cost and Utilization Project State Inpatient Databases for 14 states to identify hospital-level inpatient quality indicators (IQIs) and patient safety indicators (PSIs), by quarter and payer (Medicare vs. non-Medicare). IQIs and PSIs are standardized, evidence-based measures that can be used to track hospital quality of care and patient safety over time using hospital administrative data. The study period of 2007–2016 was selected to capture multiple years before and after introduction of program metrics. Interrupted time series was used to analyze the impact of the P4P programs on study outcomes targeted and not targeted by the programs. In sensitivity analyses, we examined the impact of these programs on care for non-Medicare patients.

**Results:**

Medicare P4P programs were not associated with consistent improvements in targeted or non-targeted quality and safety measures. Moreover, mortality rates across targeted and untargeted conditions were generally getting worse after the introduction of Medicare’s P4P programs. Trends in PSIs were extremely mixed, with five outcomes trending in an expected (improving) direction, five trending in an unexpected (deteriorating) direction, and three with insignificant changes over time. Sensitivity analyses did not substantially alter these results.

**Conclusions:**

Consistent with previous studies for individual programs, we detect minimal, if any, effect of Medicare’s hospital P4P programs on quality and safety. Given the growing evidence of limited impact, the administrative cost of monitoring and enforcing penalties, and potential increase in mortality, CMS should consider redesigning their P4P programs before continuing to expand them.

**Supplementary Information:**

The online version contains supplementary material available at 10.1186/s12913-022-08348-w.

## Background

Three major hospital pay for performance (P4P) programs were introduced by the Affordable Care Act (ACA) and intended to improve the quality, safety and efficiency of care provided to Medicare beneficiaries. The financial risk to hospitals associated with Medicare’s P4P programs is substantial. In 2019, Medicare assessed $956 million in penalties under these three programs [1] and withheld an additional 2% of inpatient payments [2] covered by the Hospital Value-Based Purchasing (HVBP) program to be used later for value-based incentive payments (penalties or bonuses).

Implemented in 2012, the Hospital Readmission Reduction Program (HRRP) penalizes hospitals for higher-than-expected readmission rates for targeted conditions (e.g., heart failure and pneumonia); hospitals may face penalties up to 3% of their Medicare revenues. That same year, the ACA also introduced the HVBP program which adjusts financial reimbursement based on specific quality, safety, and efficiency metrics including: hospital mortality, processes of care, patient safety, satisfaction, and per beneficiary spending. In 2014, the Hospital Acquired Conditions Reduction Program (HACRP) was implemented; it assesses a penalty of 1% of Medicare revenues on the worst performing quartile of hospitals each year, based on specific preventable adverse events.

Evidence on the intended impacts of these programs has been mixed. Early research suggested that introduction of the HRRP was associated with a decline in targeted readmissions [3] and larger HRRP penalties may be associated with larger improvements [3, 4]. More recent studies, however, suggest that reductions in readmissions attributed to HRRP may be overstated due to concurrent changes in electronic claim standards [5]. and regression to the mean [6]. Moreover, two recent studies, with longer follow-up data, also suggest potential unintended impact on patient mortality [7]. and a disproportionate burden on safety net providers [8] Similarly, the impact of HVBP and HACRP is not promising. Multiple studies have examined the impact of HVBP [9]; most found no impact on a wide range of targeted quality metrics, with modest evidence of improvements in pressure ulcers [10] and 30-day pneumonia mortality rates [11]. Early studies of the HACRP suggested improvements in hospital-acquired conditions, [12] but more recent studies suggest there is no clear relationships between receipt of HACRP penalties and hospital quality of care [13, 14].

Studying the combined impact of Medicare hospital P4P programs on targeted and non-targeted outcomes is important for several reasons. First, Medicare’s commitment to value-based purchasing is strong, and their policies enjoy wide bi-partisan support [15]. As a result, the Centers for Medicare and Medicaid Services (CMS) continues to expand the reach of their value-based purchasing programs, more recently implementing programs to focus on oncology care, end-stage renal disease, and the dually eligible population [16]. As P4P programs expand their reach, it is critical that we transparently examine their combined impacts on patient outcomes. Assessing the isolated impact of a single Medicare hospital P4P program is difficult since they were implemented during similar time frames. Finally, it is important to note that these three Medicare P4P programs focus a great deal of attention on a limited set of conditions and adverse events. The combined impact of this emphasis, along with potentially unintended consequences of this approach on areas and populations outside the scope of these programs, should be carefully examined. In this study, we examined the combined impact of Medicare’s P4P programs on clinical areas and populations targeted by the programs, as well as those outside their focus. While numerous outcome evaluations of the individual P4P programs have been conducted, we are not aware of any studies that examine their combined impact on targeted and untargeted patient outcomes. As CMS continues to pursue and expand P4P programs, our study offers additional evidence on the critical question of program impact.

## Methods

We combined multiple datasets to examine the combined impact of Medicare’s P4P programs on targeted and non-targeted outcomes. We used 2007–2016 Healthcare Cost and Utilization Project State Inpatient Databases (HCUP SIDs) for 14 states (Arizona, Arkansas, California, Colorado, Florida, Iowa, Kentucky, Massachusetts, Nebraska, New Jersey, New York, North Carolina, Oregon, and Washington) to identify hospital-level quality and safety outcomes, by payer (Medicare, non-Medicare). These 14 states were selected because they contained sufficient identifying information to link with other hospital- and market-level data, and they offered a sufficient volume of hospitals (> 1,000) and geographic coverage to provide meaningful insights. The cost of using data from all states in the HCUP SID was also a factor in selecting a subset of states. Data through 2016 provided at least 4 year trends after implementation of program metrics.

Table 1 provides an overview of the 14 states included in our sample. Early models included time-varying hospital- and county-level characteristics as control variables; however, including these variables induced very unsmooth (noisy) outcome trajectories and created convergence issues, so they were excluded from final models.

**Table 1:** Overview of 14 States Included in Study, Based on 2019 Data

State	Census Region	2019 Population	% Black	% Hispanic	Short Term General Hospitals
Iowa	Midwest	3,155,070	4.10%	6.30%	40
Nebraska	Midwest	1,934,408	5.20%	11.40%	27
Massachusetts	Northeast	6,892,503	9.00%	12.40%	72
New Jersey	Northeast	8,882,190	15.10%	20.90%	78
New York	Northeast	19,453,561	17.60%	19.30%	186
Arkansas	South	3,017,804	15.70%	7.80%	51
Florida	South	21,477,737	16.90%	26.40%	214
Kentucky	South	4,467,673	8.50%	3.90%	75
North Carolina	South	10,488,084	22.20%	9.80%	108
Arizona	West	7,278,717	5.20%	21.70%	77
California	West	39,512,223	6.50%	39.40%	341
Colorado	West	5,758,736	4.60%	21.80%	56
Oregon	West	4,217,737	2.20%	13.40%	37
Washington	West	7,614,893	4.40%	13.00%	61
**Total**	**---**	144,151,336	11.15%	23.55%	1,423
**US Population**		328,239,523	13.40%	18.50%	3,911

Population statistics retrieved from https://www.census.gov/quickfacts/fact/table/US/PST045219 and hospital data retrieved from https://www.ahd.com/state_statistics.html

Our primary outcome measures were hospital-level inpatient quality indicators (IQIs) and patient safety indicators (PSIs), by quarter and payer (Medicare vs. non-Medicare). IQIs and PSIs are standardized, evidence-based measures that can be used to track hospital quality of care and patient safety using hospital administrative data [17]. IQIs and PSIs were constructed using detailed algorithms available on the Agency for Healthcare Research and Quality (AHRQ) website. We chose to use IQIs and PSIs to examine the impact of the P4P programs for two reasons. First, while hospitals may focus their improvement efforts on the exact metrics targeted by CMS, we hoped to make a more global assessment of P4P program impact on hospital quality and safety. Second, we were interested in the impact of these programs on both targeted and non-targeted areas, requiring us to use a set of metrics that covered a range of conditions, not just those targeted by the P4P programs.

Table 2 provides an overview of the quality and safety measures included in our study. To investigate both intended and unintended impacts of Medicare’s P4P programs, we identified IQIs and PSIs for conditions and safety domains that were both within and outside the focus of the programs. Non-focus IQIs were further divided into clinically similar and not clinically similar conditions, either because they are for patients with a similar condition, or because they are likely to require use of similar resources or quality improvement processes within the hospital. This allowed us to identify spillover effects. Positive spillovers could occur if efforts to improve targeted domains also have a positive impact on clinically similar conditions/domains. Negative spillovers could occur if non-targeted domains worsened, or improvement trajectories attenuated, after implementation of the P4P programs.

**Table 2:** Overview of Quality and Safety Measures, by Inclusion in Medicare Pay for Performance (P4P)^a^

Quality and Safety Measures	Earliest Announcement (Implementation) of Inclusion in P4P	IQI/PSI (Study Measure)
*30-Day Mortality, Patients Included in Medicare P4P*		
AMI	FY2010^b^ (FY2013)^c^	IQI15, IQI32
HF	FY2010^b^ (FY2013)^c^	IQI16
PN	FY2010^b^ (FY2013)^c^	IQI20
Hip Replacement	FY2013 (FY2015)^c^	IQI14
*30-Day Mortality for Clinically Similar Patients*		
CABG	--	IQI12
PCI	--	IQI30
*30-Day Mortality for Not Clinically Similar Patients*		
AAA Repair	--	IQI11
Craniotomy	--	IQI13
Stroke	--	IQI17
Esophageal Resection	--	IQI18
Pancreatic Resection	--	IQI19
*Patient Safety Included in Medicare P4P*		
Composite Patient Safety	FY2012 (FY2015)^d^	PSI90^e^
*Patient Safety Not in Medicare P4P*		
Mortality among surgical patients with serious treatable complications	--	PSI14

^a^Through FY2015

^b^Announced with passage of the Affordable Care Act

^c^Area implemented first under the Hospital Readmission Reduction Program

^d^Area implemented simultaneously under both Hospital Acquired Condition Reduction Program and Hospital Value Based Purchasing

^e^Weighted average of PSI3 and PSIs 6–15; pressure ulcer, CLABSI, pneumothorax, accidental laceration, two peri-operative and five post-operative complication rates; we used the original PSI90 definition in effect during study period

We used interrupted time series to analyze the impact of Medicare’s P4P programs on study outcomes (IQIs and PSIs) targeted and untargeted by the programs. Specifically, we compared the trend in each outcome *prior to announcement* that a specific domain would be included in any of the three Medicare P4P programs with the trend in the outcome *after implementation*. Several outcomes were announced with the ACA’s passage in 2010; other announcement and implementation dates were gleaned from the Federal Register. Our approach allows for a “wash out” period between announcement and implementation to isolate impact and allow time for quality improvement efforts to have impact. To investigate whether targeted patients (Medicare) experienced different outcome trajectories from those not targeted by the P4P programs (non-Medicare), we conducted separate analyses for the two patient groups.

For inference, we used a generalized linear mixed model (GLMM) with low-rank thin plate splines (with equally spaced knots) [18] for the quarterly binomial outcomes with a logit link for each outcome type. We used binomial regression because the outcome variables were rates per quarter (e.g., the number of inpatient deaths for a particular procedure divided by inpatient discharges for that procedure). For the IQI outcomes spline, we used four knots, located at the 0.2, 0.4, 0.6, and 0.8 quantiles of the list of all quarters for all hospitals where we had a non-zero denominator for that outcome. For the spline for the PSI outcomes, because of sparse data (i.e., hospitals had zero safety events in a particular category for quarter), we reduced the number of knots from four to three, and located the knots at the 0.25, 0.5, and 0.75 quartiles.

We fitted the GLMM models using the lme4 R package [19]. Using the resulting estimates for each trajectory, we computed the change from one year prior to the announcement date to the announcement date (pre-announcement) and the change from the implementation date to one year after the implementation date (post-implementation). We computed 95% bootstrap confidence intervals for these changes (and the difference in the changes) by resampling the hospitals, refitting the GLMM, and computing the changes and difference in changes for each bootstrap sample. To assess whether our results were sensitive to trajectory specification, we also fit piecewise linear models with two change points, one each at the announcement and implementation dates, for all outcomes (Appendix).

## Results

Table 3 contains the results of our analyses for IQIs and PSIs for Medicare patients, including both focus and non-focus area measures. Notably, we find no evidence of improved IQIs for focus or non-focus areas. Trends in mortality measures are uniformly increasing or had insignificant changes in their trajectory from pre-announcement to post-implementation. Trends in PSIs (safety domains) were extremely mixed, with five outcomes trending in an expected (improving) direction, five trending in an unexpected (deteriorating) direction, and three outcomes with insignificant changes over time. Sensitivity analyses (Appendix Table A1) did not substantially alter these results.

**Table 3:** Difference Between Rate of Change^b^ in IQIs and PSIs Before Announcement and After Implementation of Metric Area (Medicare patients only)

Outcome	Description	Focus/Non-Focus^c^	Raw Trend Before Announcement^d^	Raw Trend After Implementation^e^	Rate Difference (Confidence Interval)	Expected vs. Unexpected Change
Inpatient Quality Indicators (IQIs)^a^						
IQI14	Hip Replacement Mortality^f^	focus	-0.20	0.00	0.15 (− 0.17, 0.47)	Not significant
IQI15	Acute Myocardial Infarction (AMI) Mortality	focus	-5.10	0.60	5.66 (4.12, 7.21)	Unexpected
IQI16	Heart Failure (HF) Mortality	focus	-2.60	0.50	3.11 (2.48, 3.73)	Unexpected
IQI20	Pneumonia Mortality	focus	-5.50	1.40	6.95 (6.04, 7.68)	Unexpected
IQI32	AMI w/o transfers Mortality	focus	-5.90	0.40	6.26 (4.47, 8.05)	Unexpected
IQI12	Coronary Artery Bypass Graft Mortality	non-focus, similar	-2.20	0.20	2.39 (0.74, 3.98)	Unexpected
IQI30	Percutaneous Coronary Intervention Mortality	non-focus, similar	0.90	3.20	2.25 (1.21, 3.24)	Unexpected
IQI11	Abdominal Aortic Aneurysm Repair Mortality	non-focus, not similar	-4.70	-0.30	4.37 (0.45, 8.19)	Unclear
IQI13	Craniotomy Mortality^f^	non-focus, not similar	-3.60	4.50	8.04 (4.48, 11.52)	Unclear
IQI17	Acute Stroke Mortality	non-focus, not similar	-6.00	-0.40	5.60 (4.14, 7.04)	Unclear
IQI18	Gastrointestinal Hemorrhage Mortality	non-focus, not similar	-1.10	0.00	1.16 (0.27, 1.94)	Unclear
IQI19	Hip Fracture Mortality	non-focus, not similar	-1.00	-0.70	0.25 (− 0.75, 1.28)	Not significant
Patient Safety Indicators (PSIs)^a^						
PSI03	Pressure Ulcer^g,h^	focus	-0.02	0.05	0.06 (0.05, 0.07)	Unexpected
PSI06	Iatrogenic Pneumothorax^h^	focus	-0.01	-0.04	− 0.02 (-0.03, -0.02)	Expected
PSI07	CLABSI^h,i^	focus	-0.11	-0.09	0.01 (0.00, 0.02)	Unexpected
PSI08	Inpatient Fall with Hip Fracture^h^	focus	0.00	0.00	0.001 (− 0.005, 0.007)	Not significant
PSI09	Perioperative Hemorrhage or Hematoma^j^	focus	-0.18	-0.59	-0.41 (-0.46, -0.35)	Expected
PSI10	Postop Acute Kidney Injury Requiring Dialysis^k^	focus	0.00	0.02	0.02 (-0.04, 0.07)	Not significant
PSI11	Postop Respiratory Failure^k^	focus	0.68	-1.61	-2.29 (-2.53, -2.03)	Expected
PSI12	Perioperative Pulmonary Embolism^k^	focus	-0.28	-0.12	0.16 (0.11, 0.21)	Unexpected
PSI13	Postop Sepsis^k^	focus	-0.53	-0.03	0.49 (0.33, 0.65)	Unexpected
PSI14	Postop Wound Dehiscence^l^	focus	-0.17	-0.40	-0.23 (-0.33, -0.14)	Expected
PSI15	Abdominopelvic Accidental Puncture or Laceration^l^	focus	0.03	0.05	0.02 (-0.05, 0.09)	Not significant
PSI90	Patient safety composite^m^	focus	-0.12	-0.03	0.09 (0.02, 0.12)	Unexpected
PSI04	Death of surgical patients with serious treatable complications^n^	non-focus, similar	3.40	-7.16	-10.57 (-20.94, -1.19)	Expected

^a^For additional details on definitions of IQIs and PSIs, see https://www.qualityindicators.ahrq.gov/

^b^Multiplied by 1,000 for ease of presentation

^c^Focus = clinical areas targeted by Medicare’s P4P programs; Non-focus, similar = areas not targeted but clinically similar to focus areas

^d^Rate at announcement – Rate before announcement; ^e^Rate after implementation – Rate at implementation;

^f^IQI13 and IQI14 have been retired. Reasons included limited evidence base, rare events, or practice change that affects validity

^g^Stage III or IV pressure ulcers; ^h^Per 1,000 discharges; ^i^Central line associated blood stream infections; ^j^Per 1,000 surgical discharges

^k^Per 1,000 elective surgical discharges; ^l^Per 1,000 abdominopelvic surgery discharges; ^m^Weighted average of observed-to-expected ratios for component PSIs

^n^Per 1,000 surgical discharges with serious treatable complications (deep vein thrombosis/ pulmonary embolism, pneumonia, sepsis, shock/cardiac arrest, or gastrointestinal hemorrhage/acute ulcer)

We also analyzed these same IQIs and PSIs for non-Medicare patients (Appendix Table A2), focusing especially on whether changes in metric trends pre-announcement to post-implementation in these populations were similar to what we observed for Medicare patients. We found that changes mimicked those seen for Medicare patients. The notable exception is PSI04 (Death of surgical patients with serious treatable complications) which was markedly improved for Medicare patients post-implementation, but not for non-Medicare patients in main analyses. However, in our Medicare sensitivity analysis (piecewise linear), PSI04 did not improve significantly over time.

## Discussion

We found that, in combination, Medicare’s hospital P4P programs were not associated with consistent improvements in targeted or non-targeted quality and safety measures. Moreover, mortality rates across all categories (focus, clinically similar, not clinically similar) were generally getting worse over the study period. Only one of 13 different mortality rates fell significantly after these programs were implemented (death among surgical patients with serious treatable complications; PSI04), and this result was not robust to sensitivity analysis. We did not detect improvements in mortality rates targeted by the P4P programs, nor did we detect improvements in mortality rates for clinically similar conditions.

These findings may reflect one or more factors. First, only one of the three programs (HVBP) directly targets mortality rates, and actual penalties and bonuses assessed under that program have been modest [20]. It is also possible that mortality trends are not particularly sensitive to the changes implemented by hospitals (e.g., new programs, protocols) in response to Medicare’s P4P programs, or that impact of these changes on patients or hospitals is too heterogeneous to generate a clear signal. For example, a recent study found 30-day HF mortality rates for (baseline) poor performing hospitals improved significantly over time, but mortality among all other hospitals worsened [21]. Additionally, some hospitals may respond to penalties by focusing on documentation practices rather than quality improvement activities, [22] yielding improved metrics but little impact on important outcomes like mortality. Reductions in readmissions associated with HRRP may be associated with increases in mortality [7]. Our previous research also suggests that metrics employed by Medicare’s P4P programs may be hard for hospitals to target because they are noisy (i.e. driven by random variation) [23] or updated too frequently to allow hospitals to effectively respond [24]. Whatever the root cause, our results are consistent with previous studies of Medicare P4P programs that find minimal, if any, impact on mortality [25, 26].

We found mixed evidence that Medicare’s P4P programs were associated with improved safety metrics for Medicare patients. Although not directly targeted, several components of the PSI90, including iatrogenic pneumothorax, perioperative hemorrhage, postoperative respiratory failure, and postoperative wound dehiscence, improved after implementation of the programs. However, the overall composite safety score itself, a measure included under both HACRP and HVBP, deteriorated over time for Medicare patients, driven by deteriorating trends among other component PSIs that were weighted more heavily.

It is difficult to interpret the heterogeneous patterns of improvement versus deterioration in the component PSIs. These mixed results may indicate that metric trends were driven by other factors, such as independent quality improvement programs, not by Medicare’s P4P programs. We would note, however, that two of the measures that deteriorated (pressure ulcers, CLABSIs) were already targeted by one of Medicare’s earlier P4P programs established before ACA (the Hospital Acquired Conditions Initiative). For these measures, hospitals may already have been investing in prevention, minimizing the impact of new P4P programs.

We also found that IQI and PSI trends were remarkably similar across Medicare and non-Medicare populations. This may be good news if it indicates that hospital investments to improve quality and safety also benefit similar non-Medicare patients (i.e., spillovers). However, since we did not find evidence of improved quality and safety among Medicare patients, the similarity of trends more likely supports the “no impact” narrative. In this case, the changing trends we detect may simply be driven by other time-varying factors.

We found limited evidence of unintended consequences of Medicare’s P4P programs. While several non-focus, clinically similar metrics (mortality rates for patients undergoing coronary artery bypass graft (CABG) or percutaneous coronary intervention (PCI)) worsened after implementation of P4P, the trends mirrored other IQIs for targeted conditions, which also worsened. We also observed that death rates for surgical patients with serious treatable complications, a non-targeted, clinically similar metric to other PSIs, may have improved after P4P implementation. Again, this positive trend mirrored several other targeted safety metrics. The common trends of both targeted and non-targeted metrics provide some evidence that efforts targeting particular metrics may benefit clinically similar patients.

This study has several limitations. First, because we relied on observational data and interrupted time series study design, we cannot rule out the potential influence of other, unmeasured changes that occurred during the same time frame. For example, some hospitals in our sample may have participated in accountable care organizations (ACOs), assuming greater upside and downside risk for these or similar quality and safety metrics during the study period. We also compared outcome trends prior to the announcement of a domain to after implementation; this approach may have overlooked changes occurring beyond this time frame. The inclusion of only 14 states may also limit generalizability. It is also important to note that some of the quality and safety metrics employed in our study capture relatively rare events. Modeling these rare events created informational challenges that were addressed using ensemble methods. Finally, we do not examine the impact of these P4P programs on the metrics directly targeted by the programs; it is possible that these metrics exhibited different patterns from the IQIs and PSIs we examined.

Reporting null or negative findings is always a challenge. Do our results imply Medicare’s P4P programs have a limited impact on key quality and patient safety metrics, or were we simply unable to detect the true change? Comparing our results to other empirical literature, limited impact is more likely. That is, Medicare P4P programs have not been associated with consistent improvements in quality and safety measures. Moreover, inpatient mortality rates have generally been getting worse after the introduction of Medicare’s P4P programs.

## Conclusions

We found no evidence that Medicare’s hospital P4P programs were associated with consistent improvements in quality and safety. Moreover, the mortality rates we examined were generally getting worse over the study period. Given the growing evidence of limited impact, the administrative cost of monitoring and enforcing penalties, and potential increase in mortality, CMS should consider redesigning their P4P programs before continuing to expand them.

## Supplementary Information

**Additional file 1.**

## Data Availability

The data that support the findings of this study are available for purchase from the Agency for Healthcare Research and Quality (AHRQ) through the HCUP Central Distributor. Data is available from the authors upon reasonable request and with permission from AHRQ. Programming code based on standardized HCUP SID data files is available from the authors upon reasonable request.
