# Supplementary material for: Combined impact of Medicare’s hospital pay for performance programs on quality and safety outcomes is mixed
Source: BMC Health Serv Res. 2022 Jul 28;22:958. doi: 10.1186/s12913-022-08348-w (PMC9330620; doi:10.1186/s12913-022-08348-w)
Supplement: Supplementary file 1 — Additional file 1. [file 12913_2022_8348_MOESM1_ESM.docx]

Table A1. Difference Between Rate of Change^1^ in IQIs and PSIs Before Announcement and After Implementation of Metric Area (Medicare; Piecewise Linear)

| **Outcome** | **Description** | **Focus/Non-Focus^2^** | **Raw Trend Before Announcement^3^** | **Raw Trend After Implementation^4^** | **Adjusted Difference in Rate** | **Same as Main Result** |
| --- | --- | --- | --- | --- | --- | --- |
| Inpatient Quality Indicators (IQIs)* | | | | | | |
| IQI14 | Hip Replacement Mortality^5^ | focus | -0.20 | -0.20 | 0.00 (-0.50, 0.41) | Yes |
| IQI15 | Acute Myocardial Infarction (AMI) Mortality | focus | -4.40 | 2.40 | 6.71 (3.93, 8.74) | Yes |
| IQI16 | Heart Failure (HF) Mortality | focus | -0.80 | 0.10 | 0.98 (0.04, 1.88) | Yes |
| IQI20 | Pneumonia Mortality | focus | -2.10 | 2.10 | 4.23 (3.17, 5.62) | Yes |
| IQI32 | AMI w/o transfers Mortality | focus | -4.90 | 3.30 | 8.21 (5.91, 10.93) | Yes |
| IQI12 | Coronary Artery Bypass Graft Mortality | non-focus, similar | -1.70 | -0.70 | 0.95 (-1.71, 3.48) | *Not significant* |
| IQI30 | Percutaneous Coronary Intervention Mortality | non-focus, similar | 1.70 | 5.40 | 3.80 (2.17, 5.61) | Yes |
| IQI11 | Abdominal Aortic Aneurysm Repair Mortality | non-focus, not similar | -1.80 | -2.10 | -0.27 (-7.37, 6.11) | *No* |
| IQI13 | Craniotomy Mortality^5^ | non-focus, not similar | -0.30 | 10.40 | 10.65 (5.50, 15.77) | Yes |
| IQI17 | Acute Stroke Mortality | non-focus, not similar | -4.00 | -1.50 | 2.55 (0.53, 5.38) | Yes |
| IQI18 | Gastrointestinal Hemorrhage Mortality | non-focus, not similar | -0.70 | -0.80 | -0.07 (-1.85, 1.22) | *Not significant* |
| IQI19 | Hip Fracture Mortality | non-focus, not similar | -1.20 | -1.10 | 0.04 (-2.01, 1.88) | Yes |
| Patient Safety Indicators (PSIs)* | | | | | | |
| PSI03 | Pressure Ulcer^6,7^ | focus | -0.25 | 0.06 | 0.31 (0.25, 0.36) | Yes |
| PSI06 | Iatrogenic Pneumothorax^7^ | focus | -0.01 | -0.04 | -0.04 (-0.06, -0.02) | Yes |
| PSI07 | CLABSI^7,8^ | focus | -0.14 | -0.02 | 0.12 (0.09, 0.15) | Yes |
| PSI08 | Inpatient Fall with Hip Fracture^7^ | focus | -0.04 | 0.01 | 0.04 (0.03, 0.06) | *No* |
| PSI09 | Perioperative Hemorrhage or Hematoma^9^ | focus | -0.15 | -0.82 | -0.67 (-0.83, -0.49) | Yes |
| PSI10 | Postop Acute Kidney Injury Requiring Dialysis^10^ | focus | -0.01 | -0.04 | -0.03 (-0.14, 0.08) | Yes |
| PSI11 | Postop Respiratory Failure^10^ | focus | 0.30 | -1.56 | -1.86 (-2.31, -1.36) | Yes |
| PSI12 | Perioperative Pulmonary Embolism^9^ | focus | -0.71 | -0.22 | 0.49 (0.34, 0.64) | Yes |
| PSI13 | Postop Sepsis^10^ | focus | -0.07 | 0.01 | 0.08 (-0.32, 0.35) | *Not significant* |
| PSI14 | Postop Wound Dehiscence^11^ | focus | -0.16 | -0.53 | -0.37 (-0.73, -0.05) | Yes |
| PSI15 | Abdominopelvic Accidental Puncture or Laceration^11^ | focus | 0.00 | -0.05 | -0.05 (-0.29, 0.12) | Yes |
| PSI90 | Patient safety composite^12^ | focus | -0.26 | -0.11 | 0.15 (0.00, 0.26) | Yes |
| PSI04 | Death of surgical patients with serious treatable complications^13^ | non-focus, similar | -1.69 | 16.90 | 18.59 (14.01, 23.7) | *No* |

*For additional details on definitions of IQIs and PSIs, see <https://www.qualityindicators.ahrq.gov/>

^1^Multiplied by 1,000 for ease of presentation

^2^Focus = clinical areas targeted by Medicare’s P4P programs; Non-focus, similar = areas not targeted but clinically similar to focus areas

^3^Rate at announcement – Rate before announcement; ^4^Rate after implementation – Rate at implementation;

^5^IQI13 and IQI14 have been retired. Reasons included limited evidence base, rare events, or practice change that affects validity

^6^Stage III or IV pressure ulcers; ^7^Per 1,000 discharges; ^8^Central line associated blood stream infections; ^9^Per 1,000 surgical discharges

^10^Per 1,000 elective surgical discharges; ^11^Per 1,000 abdominopelvic surgery discharges; ^12^Weighted average of observed-to-expected ratios for component PSIs

^13^Per 1,000 surgical discharges with serious treatable complications (deep vein thrombosis/ pulmonary embolism, pneumonia, sepsis, shock/cardiac arrest, or gastrointestinal hemorrhage/acute ulcer)

Table A2. Difference Between Rate of Change^1^ in IQIs and PSIs Before Announcement and After Implementation of Metric Area (Non-Medicare patients only)

| **Outcome** | **Description** | **Focus/Non-Focus^2^** | **Raw Trend Before Announcement^3^** | **Raw Trend After Implementation^4^** | **Adjusted Difference in Rate** | **Change Similar to Medicare (Yes/No)** |
| --- | --- | --- | --- | --- | --- | --- |
| Inpatient Quality Indicators (IQIs)* | | | | | | |
| IQI14 | Hip Replacement Mortality^5^ | focus | -0.10 | 0.00 | 0.12 (-0.06, 0.30) | Not significant |
| IQI15 | Acute Myocardial Infarction (AMI) Mortality | focus | -3.90 | 0.90 | 4.84 (3.62, 6.04) | Yes |
| IQI16 | Heart Failure (HF) Mortality | focus | -2.60 | 0.50 | 3.04 (2.51, 3.60) | Yes |
| IQI20 | Pneumonia Mortality | focus | -4.50 | 1.40 | 5.91 (5.28, 6.50) | Yes |
| IQI32 | AMI w/o transfers Mortality | focus | -4.70 | 0.80 | 5.44 (4.15, 6.77) | Yes |
| IQI12 | Coronary Artery Bypass Graft Mortality | non-focus, similar | -1.70 | 0.30 | 2.06 (1.03, 3.04) | Yes |
| IQI30 | Percutaneous Coronary Intervention Mortality | non-focus, similar | 1.10 | 2.20 | 1.12 (0.46, 1.80) | Yes |
| IQI11 | Abdominal Aortic Aneurysm Repair Mortality | non-focus, not similar | -3.80 | -0.50 | 3.32 (-0.13, 6.71) | Yes |
| IQI13 | Craniotomy Mortality^5^ | non-focus, not similar | -4.00 | 4.10 | 8.02 (5.83, 10.03) | Yes |
| IQI17 | Acute Stroke Mortality | non-focus, not similar | -5.50 | -0.40 | 5.03 (3.85, 6.17) | Yes |
| IQI18 | Gastrointestinal Hemorrhage Mortality | non-focus, not similar | -0.90 | 0.20 | 1.02 (0.40, 1.66) | Yes |
| IQI19 | Hip Fracture Mortality | non-focus, not similar | -1.10 | -0.50 | 0.57 (-0.39, 1.59) | Yes |
| Patient Safety Indicators (PSIs)* | | | | | | |
| PSI03 | Pressure Ulcer^6,7^ | focus | 0.00 | 0.04 | 0.04 (0.03, 0.05) | Yes |
| PSI06 | Iatrogenic Pneumothorax^7^ | focus | -0.01 | -0.03 | -0.02 (-0.03, -0.02) | Yes |
| PSI07 | CLABSI^7,8^ | focus | -0.10 | -0.08 | 0.02 (0.01, 0.02) | Yes |
| PSI08 | Inpatient Fall with Hip Fracture^7^ | focus | 0.00 | 0.00 | 0.00 (0.00, 0.00) | Yes |
| PSI09 | Perioperative Hemorrhage or Hematoma^9^ | focus | -0.15 | -0.52 | -0.37 (-0.41, -0.33) | Yes |
| PSI10 | Postop Acute Kidney Injury Requiring Dialysis^10^ | focus | 0.02 | 0.01 | 0.00 (-0.04, 0.03) | Not significant |
| PSI11 | Postop Respiratory Failure^10^ | focus | 0.66 | -1.09 | -1.75 (-1.91, -1.60) | Yes |
| PSI12 | Perioperative Pulmonary Embolism^9^ | focus | -0.19 | -0.03 | 0.16 (0.13, 0.19) | Yes |
| PSI13 | Postop Sepsis^10^ | focus | -0.28 | 0.01 | 0.29 (0.20, 0.38) | Yes |
| PSI14 | Postop Wound Dehiscence^11^ | focus | -0.07 | -0.29 | -0.21 (-0.27, -0.16) | Yes |
| PSI15 | Abdominopelvic Accidental Puncture or Laceration^11^ | focus | 0.04 | 0.08 | 0.04 (-0.01, 0.08) | Not significant |
| PSI90 | Patient safety composite^12^ | focus | -0.03 | 0.05 | 0.07 (0.02, 0.09) | Yes |
| PSI04 | Death of surgical patients with serious treatable complications^13^ | non-focus, similar | 0.33 | 8.60 | 8.26 (7.08, 9.34) | No |

*For additional details on definitions of IQIs and PSIs, see <https://www.qualityindicators.ahrq.gov/>

^1^Multiplied by 1,000 for ease of presentation

^2^Focus = clinical areas targeted by Medicare’s P4P programs; Non-focus, similar = areas not targeted but clinically similar to focus areas

^3^Rate at announcement – Rate before announcement; ^4^Rate after implementation – Rate at implementation;

^5^IQI13 and IQI14 have been retired. Reasons included limited evidence base, rare events, or practice change that affects validity

^6^Stage III or IV pressure ulcers; ^7^Per 1,000 discharges; ^8^Central line associated blood stream infections; ^9^Per 1,000 surgical discharges

^10^Per 1,000 elective surgical discharges; ^11^Per 1,000 abdominopelvic surgery discharges; ^12^Weighted average of observed-to-expected ratios for component PSIs

^13^Per 1,000 surgical discharges with serious treatable complications (deep vein thrombosis/ pulmonary embolism, pneumonia, sepsis, shock/cardiac arrest, or gastrointestinal hemorrhage/acute ulcer)
